# Evaluation of Scheimpflug imaging parameters in blepharospasm and normal eyes

**DOI:** 10.1186/s12886-018-0897-9

**Published:** 2018-09-05

**Authors:** Huina Zhang, Hongjie Zhou, Tiepei Zhu, Juan Ye

**Affiliations:** 1 Department of Ophthalmology, the Second Affiliated Hospital, Zhejiang University School of Medicine, Hangzhou, Zhejiang China; 2 Hangzhou Hospital for the Prevention and Treatment of Occupational Diseases, Hangzhou, Zhejiang China

**Keywords:** Blepharospasm, Pentacam, Corneal curvature Corneal topography

## Abstract

**Background:**

To investigate changes in corneal elevation, pachymetry, and keratometry in discriminating between normal and blepharospasm eyes, as measured by the Pentacam rotating Scheimpflug camera.

**Methods:**

This was a prospective, cross-sectional study. A total of 47 consecutive patients with a range of blepharospasm severity and 40 age- and sex- matched healthy subjects were included, one eye of each subject was randomly chosen for data analysis. Blepharospasm severity was evaluated using the Jankovic scale and categorized as mild, moderate, or severe. Corneal parameters were measured by the Pentacam rotating Scheimpflug camera to derive corneal tomography information. Various parameters regarding keratometry, elevation at the anterior and posterior corneal surface, pachymetric data, final D value, and topometric indices from the Pentacam software were recorded, and the relationship between the blink rate and corneal parameters was analyzed. Intraclass correlation coefficients (ICCs) were assessed to evaluate the repeatability of intraobserver.

**Results:**

Increased topographic asymmetry was observed in moderate and severe blepharospasm. Front K1and front Km were significantly higher in cases of mild (*P* < 0.05), moderate (*P* < 0.0001), and severe (*P* < 0.0001) blepharospasm as compared with controls. Front K2, back K1, back K2, and back Km were significantly higher in cases of moderate (*P* < 0.01) and severe (*P* < 0.001) blepharospasm as compared with controls. For corneal topometric indices, both ISV and IVA were significantly increased in severe blepharospasm (*P* < 0.05). Radii minimum were significantly increased in cases of moderate and severe blepharospasm (*P* < 0.05).There were no differences in corneal elevation and corneal pharcymetric parameters among the four groups, except for front BFS, which was significantly different in blepharospasm groups (*P* < 0.05). Final D values were significantly higher in the severe blepharospasm (*P* < 0.01) group than that among controls. There were significant correlations between the blink rate and most corneal tomographic parameters. All parameters showed high reproducibility (ICC: 0.921–0.996) for normal and blepharospasm subjects.

**Conclusions:**

Blepharospasm may lead to a redistribution of the pressure applied by the lids over the cornea and, consequently, may result in corneal shape changes, which can be documented through corneal topography.

## Background

Benign essential blepharospasm is one of the most common neuromuscular disorders. In cases of blepharospasm, a grumbling facial expression, the fluttering of the eyelids, an increased frequency of blinking, and chronic involuntary contractions are the main signs [1].It is well-known that the corneal surface is susceptible to eyelid pressure [2–4].Moon et al. [5] reported corneal astigmatism changes in patients with blepharospasm or hemifacial spasm after the injection of botulinum toxin (BTX). Thus, in addition to the cosmetic problems involved in eyelid appearance, blepharospasm may also lead to a redistribution of the pressure applied by the lids over the cornea and, consequently, may result in corneal shape changes, which can be documented through corneal topography.

Placido disk-based corneal topography and biomicroscopic examination are widely used in the clinical diagnosis of cornea diseases. However, the corneal topography study only yields the measurement of anterior corneal surface and cannot reflect the alteration of the entire corneal architecture. It has been shown that corneal curvature measurements performed via the Pentacam have excellent repeatability [6]. The Pentacam (Oculus, Inc., Wetzlar, Germany) is a piece of equipment that uses a rotating Scheimpflug camera to image the anterior segment (including both the anterior and posterior corneal surfaces) [7, 8].It measures 25,000 data points from 50 meridians over the cornea in less than 2 s [9]. Although previous research using Scheimpflug photography has investigated astigmatism of the posterior corneal surface, the curvature of the cornea was only measured along 6 meridians [10]. The magnitude and axis of the astigmatism obtained in these studies may not be as accurate as those obtained via the Pentacam.

To the best of our knowledge, no studies have been conducted regarding corneal parameter differences between normal and blepharospasm eyes, as measured by Scheimpflug imaging. Therefore, the aim of present study is to prospectively determine the efficacy of corneal elevation, pachymetry, and keratometry in discriminating between normal and blepharospasm eyes with respect to Jankovic Rating scale stage [11],with a view to contributing to our understanding of the specific corneal structural alterations that occur in blepharospasm.

## Methods

### Subjects and clinical evaluations

This prospective, case-control study included 47 patients with blepharospasm (10 male and 37 female eyes, mean age: 58.64 ± 9.03 years old) and 40 normal subjects (candidates for cataract surgery with normal corneas, 13 male and 27 female eyes, mean age: 59.18 ± 10.05 years old), one eye of each subject was randomly chosen for data analysis. Before study enrollment, all patients provided informed consent to participate in the research. The research protocol followed the tenets of the Declaration of Helsinki and was approved by the ethics committee of the Second Affiliated Hospital, Zhejiang University School of Medicine, from August 2016 through December 2016.A diagnosis of blepharospasm was established via the Jankovic Rating scale [12] (0 = no spasm; 1 = mild spasm, barely noticeable; 2 = mild spasm, without functional impairment; 3 = moderate spasm, with moderate functional impairment; 4 = severe, incapacitating spasm), categorizing the eyes as mild(12 eyes), moderate(20 eyes), or severe(15 eyes). All patients must have had the symptoms of blepharospasm for over six months according to this classification system.

Ophthalmic examinations consisted of best-corrected visual acuity measurements, slit-lamp examination, and extraocular movements. Patients with the following conditions were excluded from the study: a history of ocular or eyelid surgery; glaucoma; blepharoptosis; strabismus; significant hyperopia (> + 1 diopter); corneal abnormalities due to other factors, such as trauma, keratoconus, chronic eye rubbing, and vernal keratoconjunctivitis; and anyone who was unable to cooperate with the examinations. The participants who wore soft contact lenses were asked to stop for at least 2 weeks,and those who wore rigid contact lenses were asked to stop using them for 5 weeks before this assessment.

The blink rate was measured via direct observation [13]. Two investigators sat in the room during a lecture and secretly counted the blinks of each subject for 1 min using a mechanical counter, repeated for two times. The data were pooled, and the results were expressed as the means±standard deviations based on four independent experiments. Subjects were unaware of the blink measurements.

### Corneal tomography

We followed the methods of Zhu et al.2017 [14]. A Pentacam HR system (Oculus, Wetzlar, Germany) was used to evaluate the anterior and posterior corneal surfaces. The measurements were performed in the automatic release mode by the same experienced examiner, and 25 rotating Scheimpflug images were obtained for each eye within 2 s. Image quality was checked, and for each eye, only one high-quality examination was recorded. The sagittal curvature, front elevation, corneal thickness, back elevation, and Belin/Ambrósio Enhanced Ectasia Display were evaluated. Elevation data were measured in a standardized fashion relative to a reference best-fit sphere (BFS) that was calculated at a fixed optical zone of 8.0 mm.

The following data were obtained with this instrument: (1) keratometric values: flat keratometry (K1), steep keratometry (K2), mean keratometry (Km), astigmatism altitude, and axis for the central 3.0 mm of cornea;(2) topometric indices: index of surface variance (ISV), index of vertical asymmetry (IVA), keratoconus-index (KI), center keratoconus-index (CKI), index of height decentration (IHD), index of height asymmetry (IHA), and radii minimum (RM); (3) variables in elevation map: diameter of BFS, elevation at apex point, maximum elevation, and elevation at the thinnest point within the central 4.0 mm zone; (4) corneal pachymetric parameters: corneal thickness at the thinnest point and at the apex, the difference of thickness between these two points (apex/thinnest difference), the thinnest location, pachymetric progression indices, and Ambrósio’s relational thickness (ART); and (5) final D-value.

The astigmatism value of both corneal surfaces was converted into the rectangular forms of Fourier notation (J0 [Jackson cross-cylinder with axes at 180° and 90°] and J45 [Jackson cross-cylinder with axes at 45° and 135°]) for power vector analysis using the following equations: J0 = (-C/2)sin2α and J45 = (-C/2) cos2α, where C was the corneal astigmatism magnitude, and α was the meridian of steep keratometry [15].

### Statistical analysis

Statistical analysis was performed with a one-way ANOVA, followed by the Dunnett multiple-comparisons test (GraphPad Prism 5 software; GraphPad Software, San Diego, CA). A *p*-value of < 0.05 was considered to be statistically significant. All results were compared between investigators by using the intraclass correlation coefficient (ICC). Results from one investigator were reported in this study as the mean and standard deviation for all eyes. Additionally, spearman correlation analyses were used to define the correlation between the blink rate and Pentacam parameters. All statistical analyses were performed using the Statistical Package for the Social Science, Version 20.0 (SPSS Inc., Chicago, IL). A *p*-value of < 0.05 was considered statistically significant.

## Results

The characteristics of the study subjects are presented in Table 1. There were no age-, disease duration-, or sex-related statistical differences between patients with blepharospasm and control subjects. The average blink rate was significantly increased in each blepharospasm subgroup (*p* < 0.0001).

**Table 1:** Comparison of baseline characteristics between control and blepharospasm groups

	Control	Blepharospasm		
		*Mild*	*Moderate*	*Severe*
Age (yrs)	59.18 ± 10.05	54.07 ± 8.67	60.05 ± 8.51	60.4 ± 9.32
Male (n, %)	13 (32.5%)	4 (33.3%)	5 (25%)	2 (13.3%)
Subjects (n)	40	12	20	15
Jankovic Rating Scale	0	1 or 2	3	4
Disease duration (mean yrs. + SD)	N	3.9 ± 2.7	4.4 ± 3.2	3.4 ± 3.2
Blink Rate (blinks/min)	17.56 ± 3.62	30.64 ± 5.41****	39.54 ± 5.99****	52.45 ± 7.79****

Jankovic Rating Scale: 0 = no spasm; 1 = mild spasm, barely noticeable; 2 = mild spasm, without functional impairment; 3 = moderate spasm, with moderate functional impairment;4 = severe, incapacitating spasm

Data are mean standard deviation unless otherwise indicated

****P < 0.0001, versus controls

### Keratometric parameters

Table 2 provides the keratometric parameters in the blepharospasm and control groups. Front K1and front Km were significantly higher in cases of mild (*p* < 0.05), moderate (*p* < 0.0001), and severe (*p* < 0.0001) blepharospasm. Front K2, back K1, back K2, and back Km were significantly higher in cases of moderate (*p* < 0.01) and severe (*p* < 0.001) blepharospasm. However, there were no significant differences among the four groups in terms of front J0, front J45, front astigmatism magnitude (front Astig), back J0, back J45, and back Astig.

**Table 2:** Comparison of keratometric parameters between control and blepharospasm groups

	Control	Blepharoptosis		
		*Mild*	*Moderate*	*Severe*
Front K1 (D)	42.24 ± 1.26	43.28 ± 0.89*	44.58 ± 1.42****	44.51 ± 1.37****
Front K2 (D)	43.32 ± 1.41	43.96 ± 1.35	44.86 ± 1.83***	45.55 ± 1.09****
Front Km (D)	42.82 ± 1.32	43.94 ± 0.86*	44.56 ± 1.77****	44.99 ± 1.05****
Front Astig (D)	1.21 ± 0.60	1.03 ± 0.57	0.81 ± 0.30	1.05 ± 1.38
Front J0 (D)	0.05 ± 0.39	0.09 ± 0.46	− 0.06 ± 0.22	0.18 ± 0.52
Front J45 (D)	0.01 ± 0.42	− 0.10 ± 0.38	−0.02 ± 0.25	0.11 ± 0.67
Back K1 (D)	− 6.04 ± 0.22	− 6.03 ± 0.15	− 6.36 ± 0.29****	−6.34 ± 0.24***
Back K2 (D)	− 6.37 ± 0.26	−6.44 ± 0.19	−6.61 ± 0.29**	−6.68 ± 0.14***
Back Km (D)	− 6.19 ± 0.23	−6.22 ± 0.15	−6.48 ± 0.29****	−6.50 ± 0.14****
Back Astig(D)	0.32 ± 0.16	0.38 ± 0.13	0.26 ± 0.11	0.32 ± 0.23
Back J0 (D)	0.03 ± 0.15	− 0.08 ± 0.10	−0.03 ± 0.09	0.02 ± 0.16
Back J45 (D)	−0.01 ± 0.09	−0.02 ± 0.13	−0.002 ± 0.10	−0.02 ± 0.12

*Astig* astigmatism magnitude, *D* diopter

Data are mean standard deviation unless otherwise indicated

*p < 0.05, **P < 0.01, ***P < 0.001 and ****P < 0.0001 versus controls

### Corneal topometric indices

Among corneal topometric indices, both ISV and IVA were significantly increased in cases of severe blepharospasm (*p* < 0.05). Radii minimum were significantly increased in cases of moderate and severe blepharospasm (*p* < 0.05). No significant changes in keratoconus index, central keratoconus index, index of height asymmetry or index of height decentration were noted among the four groups (P>0.05) (Table 3).

**Table 3:** Comparison of corneal topometric indices between control and blepharospasm

	Control	Blepharospasm		
		*Mild*	*Moderate*	*Severe*
Index of surface variance	20.9 ± 13.4	20.83 ± 11.5	18.1 ± 5.26	29.67 ± 11.7*
Index of vertical asymmetry	0.16 ± 0.10	0.16 ± 0.05	0.15 ± 0.06	0.23 ± 0.09*
Keratoconus index	1.02 ± 0.03	1.02 ± 0.02	1.02 ± 0.03	1.04 ± 0.03
Central keratoconus index	1.002 ± 0.01	1.01 ± 0.01	1.001 ± 0.01	1.007 ± 0.01
Index of height asymmetry	6.13 ± 4.69	7.07 ± 5.28	6.05 ± 5.58	9.46 ± 8.42
Index of height decentration	0.01 ± 0.01	0.01 ± 0.01	0.01 ± 0.01	0.02 ± 0.01
Radii minimum	7.63 ± 0.64	7.32 ± 0.15	7.31 ± 0.37*	7.18 ± 0.20**

Data are mean standard deviation unless otherwise indicated

*P < 0.05, ** *P* < 0.01, versus controls

### Corneal elevation and corneal pharcymetric parameters

In the elevation maps, no statistically significantly differences between the variables were noted among the four groups in terms of elev front apex, elev front thinnest, elev front max 4.0 mm, elev back apex, elev back thinnest, elev back max 4.0 mm, and back BFS, whereas front BFS diameter was significantly lower in blepharospasm groups than in the control group (*p* < 0.05, Table 4).

**Table 4:** Comparison of corneal elevation parameters between control and blepharospasm

	Control	Blepharoptosis		
		*Mild*	*Moderate*	*Severe*
Elev front apex (μm)	1.48 ± 1.43	1.17 ± 1.12	1.10 ± 1.59	1.87 ± 1.77
Elev front thinnest (μm)	1.05 ± 2.56	0.42 ± 1.44	1.10 ± 1.59	1.40 ± 2.13
Elev front max 4.0 mm (μm)	4.74 ± 2.99	3.08 ± 0.79	3.90 ± 2.12	4.47 ± 2.50
Front BFS (mm)	7.82 ± 0.24	7.62 ± 0.16*	7.62 ± 0.31**	7.58 ± 0.12**
Elev back apex (μm)	2.68 ± 2.52	3.25 ± 4.73	1.90 ± 2.53	2.87 ± 2.56
Elev back thinnest (μm)	5.08 ± 3.50	4.67 ± 4.29	4.70 ± 3.37	5.40 ± 4.31
Elev back max 4.0 mm (μm)	9.90 ± 4.30	9.25 ± 3.93	9.00 ± 4.11	8.47 ± 4.45
Back BFS (mm)	6.45 ± 0.39	6.41 ± 0.12	6.29 ± 0.30	6.26 ± 0.12

BFS = diameter of best fit sphere in 8.0-mm area; Elev = elevated; Max = maximum; mm = millimeter; μm = micrometer

Data are mean standard deviation unless otherwise indicated

*P < 0.05, **P < 0.01, versus controls

As illustrated in Table 5,the corneal pharcymetric parameters in the eyes with blepharospasm, including apex thickness, thinnest thickness, apex/thinnest difference, thinnest location, min PI, max PI, average PI, max ART, and average ART, did not differ from those of controls (*P* >0.05, Table 5).

**Table 5:** Comparison of corneal pharcymetric parameters between control and blepharospasm

	Control	Blepharoptosis		
		*Mild*	*Moderate*	*Severe*
Apex thickness (μm)	542.2 ± 35.59	567.6 ± 28.46	542.6 ± 28.37	543.9 ± 36.10
Thinnest thickness (μm)	537.8 ± 34.82	561.4 ± 29.31	536.3 ± 26.85	538.8 ± 37.60
Apex/Thinnest difference (μm)	5.20 ± 5.15	4.17 ± 3.19	6.25 ± 5.63	5.13 ± 3.81
Thinnest location (mm)	0.71 ± 0.31	0.72 ± 0.24	0.92 ± 0.69	0.79 ± 0.34
Min PI	0.71 ± 0.19	0.57 ± 0.22	0.59 ± 0.65	0.71 ± 0.19
Max PI	1.33 ± 0.25	1.13 ± 0.15	1.82 ± 2.63	1.46 ± 0.73
Avg PI	1.00 ± 0.15	0.90 ± 0.10	0.94 ± 0.24	0.93 ± 0.31
Max ART (μm)	424.2 ± 92.24	483.8 ± 55.06	437.9 ± 39.00	448.9 ± 114.8
Avg ART (μm)	548.0 ± 98.61	621.3 ± 104.8	545.0 ± 60.51	571.1 ± 122.4

*ART* Ambrósio’s relational thickness, *Avg* average, *Max* maximum, *Min* minimum, *mm* millimeter, *PI* progression index, *μm* micrometer

Data are mean standard deviation unless otherwise indicated

### Final D,the correlations of blink rate with corneal parameters and ICC

**Fig. 1:**
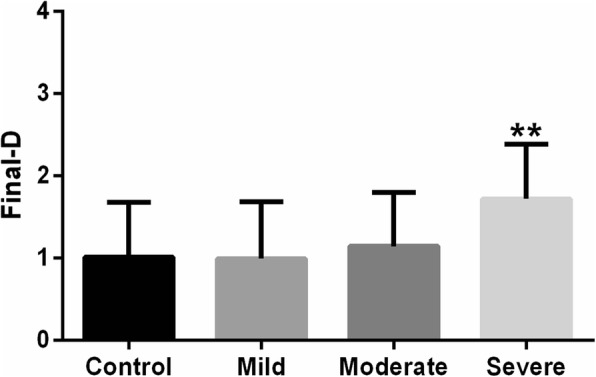
The distribution of final D values in healthy controls and blepharospasm groups. The final D value increased with increasing severity of blepharospasm. *p < 0.05, one-way ANOVA with the Dunnett’s multiple comparison test

**Fig. 2:**
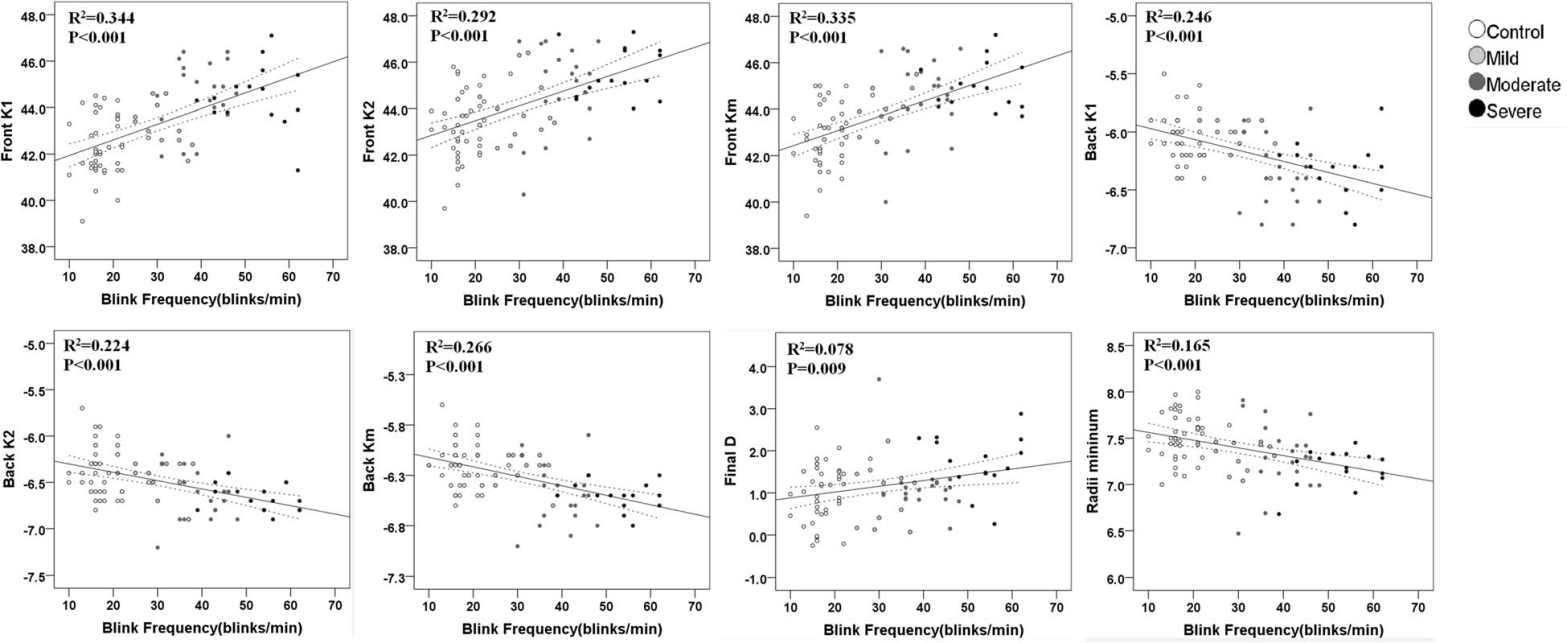
Correlations between blink rate (blinks/min) and corneal parameters, including front K1, front K2, front Km, back K1, back K2, back Km, final D and radii minimum. Spearman rank correlation coefficients (R^2^ value) are shown with statistical significance of the correlations. The linear regression line is shown with the 95% confidence intervals of mean

As shown in Fig. 1, the mean final D values were significantly higher in the severe blepharospasm (1.72 ± 0.67, *p* < 0.01) group than among controls (1.01 ± 0.66). Figure 2 shows the correlations between blink rate with corneal parameters. Blink rate was significantly and negatively correlated with back K1 (R^2^ = 0.246, *p* < 0.001), back K2 (R^2^ = 0.224, *p* < 0.001), back Km (R^2^ = 0.266, *p* < 0.001), and RM (R^2^ = 0.165, *p* < 0.001). A significant positive correlation was also found between blink rate and front K1 (R^2^ = 0.344, *p* < 0.001), front K2 (R^2^ = 0.292, *p* < 0.001), front Km (R^2^ = 0.335, *p* < 0.001) and final D (R^2^ = 0.078, *p* = 0.009). Table 6 summarises ICC, indicating that corneal curvature measurements performed via the Pentacam had good repeatability (ICC: 0.921–0.996).

**Table 6:** Intraclass correlation coefficient of parameters between control and blepharospasm

	Control	Blepharoptosis		
		*Mild*	*Moderate*	*Severe*
Anterior				
Keratometric (K1, K2, Km)	0.991–0.994	0.984–0.998	0.992–0.996	0.981–0.992
Topometric (ISV, IVA, KI, CKI, IHD, IHA)	0.982–0.989	0.972–0.994	0.987–0.994	0.946–0.978
Elevation	0.975–0.985	0.964–0.991	0.943–0.965	0.923–0.967
Posterior				
Keratometric (K1, K2, Km)	0.967–0.995	0.981–0.995	0.942–0.987	0.921–0.965
Elevation	0.932–0.985	0.946–0.979	0.921–0.954	0.934–0.981
Pachymetry				
Pachymetry parameters	0.945–0.967	0.945–0.967	0.923–0.956	0.943–0.956
(Apex thickness, Min PI, Max PI)	0.978–0.981	0.954–0.976	0.925–0.975	0.932–0.967

*K1* flat keratometry, *K2* steep keratometry, *Km* mean keratometry, *ISV* index of surface variance, *IVA* index of vertical asymmetry, *KI* keratoconus-index, *CKI* center keratoconus-index, *IHD* index of height decentration, *IHA* index of height asymmetry, *PI* progression index

## Discussion

The corneal surface is vulnerable to eyelid pressure [2]. Using the Pentacam system, Zhu et al. [14] indicated that congenital blepharoptosis not only induced corneal asymmetry and irregularity, but also affected corneal tomography, such as increased corneal elevation in blepharoptosis eyes with more than moderate severity and even focalized corneal thinning in severe cases. Changes in corneal topography and corneal astigmatism induced by eyelid surgeries were also observed after gold-weight implant, ptosis, ectropion, and eyelid mass surgeries [16, 17].Such changes may be explained by the anatomical proximity between the eyelids and the cornea and by the pressure exerted by the eyelids on the corneal surface, resulting in corneal deformation [17].

There have been relatively few reports in the literature on the effect of blepharospasm on corneal curvatures. Using corneal topography, Osaki has demonstrated that patients treated with BTX-A for hemifacial spasm developed significant eyelid and corneal changes on the affected eyes during the toxin’s period of action. The results showed a statistically significant decrease in steep K and astigmatism 2 months after BTX-A treatment [18]. In the present study, a significant difference in corneal astigmatism magnitude was found between the control and blepharospasm groups, including front K1, front K2, front Km, back K1, back K2, and back Km, and the curvature change is positively proportional to the severity of the disease. Additionally, we found the radii minimum were significantly decreased in the moderate and severe groups, indicating the decrease of the smallest radius of curvature in the entire field of measurements, further confirming corneal curvature change in the blepharospasm groups.

The Pentacam system could also provide several topometric indices that only consider the anterior corneal surface. These changes may be related to either the restoration of symmetry in the upper and lower lid apposition on the cornea or the rearrangement of the tear film [17]. We found a significant difference between ISV and IVA in the severe group. These results indicate that blepharospasm may increase the asymmetry and irregularity of the anterior corneal surface in cases of severe blepharospasm.

Among the properties of the corneal surface, elevation provides the most accurate representation of its shape. However, the Pentacam system have two reference database in Belin/Ambrósio Enhanced Ectasia Display, including Myopia/Normal and Hyperopia/Mixed Cyl, previous clinical observations indicated that there is an increased variability in the posterior elevation in hyperopic eyes on tomographic evaluation [19]. If this is true, it would lead to false positives when compared against a myopic biased normative database. Therefore, we exclude patients with significant hyperopia (> + 1 diopter). Corneal topographers can be categorized into two groups based on whether they can measure the elevation of the anterior and posterior surfaces of the cornea (front BFS and back BFS). However, to our knowledge, no study has reported on changes in corneal elevation in blepharospasm eyes. In our study, no significant difference in most corneal elevation parameters was found between the control and blepharospasm groups, except for front BFS, which was significantly different in the severe group. The diagnostic value of front BFS was suggested by Lim et al. [20], who found that front BFS was significantly higher among cases of keratoconus. However, to our knowledge of the seindices, their variability, as well as their value in reflecting the shape of the cornea are limited, and much research is still required before they can be applied with confidence and certainty.

An increased frequency of blinking was one of the main symptoms of blepharospasm. The normal blink rate is about 12 blinks/min [21], but a mean blink rate of 24.8 blinks/min has also been reported [22]. In the present study, the normal blink rate was about 17.56 ± 3.62blinks/min, while blepharospasm patients had rates of 30.64 ± 5.41blinks/min or higher. Rapid blinking is associated with worse ocular surface disease and tear stability [23], and we found a correlation between blink rate and several corneal parameters, including front K1, front K2, front Km, back K1, back K2, back Km, final D, and radii minimum, indicating that rapid blinking also increases the mechanical pressure on the surface of the cornea and changes the corneal curvature.

The final D index from the Belin/Ambrósio Enhanced Ectasia Display is amultimetric combination parameter composed of keratometric, pachymetric, pachymetric progression, and back elevation parameters. It is suggested that the final D index could be used as the sole parameter to identify early corneal ectasia [24]. Using a final D value greater than 2.61 as a cut off value may help to identify the majority of keratoconus suspects who truly have the disease [25]. In the present study, we found that final D values were significantly higher in the severe blepharospasm group than among controls, which indicated a high risk of subclinical keratoconus-like changes in severely blepharospastic eyes.

The limitations of this study should be noted as well. Firstly, all included subjects were Chinese. Because the anatomy of the eyelids and orbits differ between Asians and other races, the accurate definition and classification of blepharospasm also differ between these groups [26, 27]. Secondly, the sample size of our study was small, and the parameters must be investigated in a larger patient group. Third, it is a single-center study, which may make our results prone to a hospital-based bias. Finally, it is important to be aware of the predictive limitations of our cross-sectional study. Although the cross-sectional design allowed us to provide evidence of corneal tomography differences between blepharospasm patients and the control group, longitudinal design studies are necessary to establish a true cause-and-effect relationship.

## Conclusions

In summary, this study measured the corneal architecture with a Pentacam rotating Scheimpflug camera, and proved that the pressure applied by the lids over the cornea may result in corneal shape changes in blepharospasm patients, particularly in severe cases, which may play a critical role in everyday diagnostic procedures and the pre-operative screening of patients seeking refractive surgery.

## Abbreviations

ART: Ambrósio’s relational thickness
BFS: Best-fit sphere
BTX: Botulinum toxin
CKI: Center keratoconus-index
Front Astig: Front astigmatism magnitude
IHA: Index of height asymmetry
IHD: Index of height decentration
ISV: Index of surface variance
IVA: Index of vertical asymmetry
K1: Flat keratometry
K2: Steep keratometry
KI: Keratoconus-index
Km: Mean keratometry
PI: Progression index
RM: Radii minimum
